# Head-to-head comparison of left ventricular strain assessed by CMR post-processing tools and fast strain-ENCoded imaging ^†^

**DOI:** 10.1093/ehjimp/qyag014

**Published:** 2026-01-23

**Authors:** Andreas Ochs, Marc Zahlten, Janek Salatzki, Lukas D Weberling, James G Whayne, Christian Stehning, Evangelos Giannitsis, Claudia M Denkinger, Uta Merle, Sebastian Buss, Norbert Frey, Henning Steen, Florian André

**Affiliations:** Department of Cardiology, Angiology, and Pneumology, University Hospital Heidelberg, Im Neuenheimer Feld 410, 69120 Heidelberg, Germany; DZHK (German Centre for Cardiovascular Research), Partner Site Heidelberg, Heidelberg, Germany; Department of Cardiology, Angiology, and Pneumology, University Hospital Heidelberg, Im Neuenheimer Feld 410, 69120 Heidelberg, Germany; Department of Cardiology, Angiology, and Pneumology, University Hospital Heidelberg, Im Neuenheimer Feld 410, 69120 Heidelberg, Germany; DZHK (German Centre for Cardiovascular Research), Partner Site Heidelberg, Heidelberg, Germany; Department of Cardiology, Angiology, and Pneumology, University Hospital Heidelberg, Im Neuenheimer Feld 410, 69120 Heidelberg, Germany; DZHK (German Centre for Cardiovascular Research), Partner Site Heidelberg, Heidelberg, Germany; Myocardial Solutions Inc., Morrisville, NC, USA; Philips Healthcare, Hamburg, Germany; Department of Cardiology, Angiology, and Pneumology, University Hospital Heidelberg, Im Neuenheimer Feld 410, 69120 Heidelberg, Germany; DZHK (German Centre for Cardiovascular Research), Partner Site Heidelberg, Heidelberg, Germany; Division of Infectious Disease and Tropical Medicine, University Hospital Heidelberg, Heidelberg, Germany; German Center of Infection Research, Partner Site Heidelberg, Heidelberg, Germany; Department of Gastroenterology, Hepatology and Infectious Diseases, University Hospital Heidelberg, Heidelberg, Germany; Das Radiologische Zentrum, Heidelberg, Germany; Department of Cardiology, Angiology, and Pneumology, University Hospital Heidelberg, Im Neuenheimer Feld 410, 69120 Heidelberg, Germany; DZHK (German Centre for Cardiovascular Research), Partner Site Heidelberg, Heidelberg, Germany; Department of Cardiology, Angiology, and Pneumology, University Hospital Heidelberg, Im Neuenheimer Feld 410, 69120 Heidelberg, Germany; medneo, Hamburg, Germany; Department of Cardiology, Angiology, and Pneumology, University Hospital Heidelberg, Im Neuenheimer Feld 410, 69120 Heidelberg, Germany; DZHK (German Centre for Cardiovascular Research), Partner Site Heidelberg, Heidelberg, Germany

**Keywords:** left ventricular strain, feature tracking, post-processing strain, fast strain-ENCoded imaging, CMR

## Abstract

**Aims:**

Cardiovascular magnetic resonance (CMR) strain imaging allows early detection of subclinical myocardial dysfunction and provides incremental diagnostic and prognostic information. Strain can be derived from dedicated sequences such as fast Strain-ENCoded imaging (fSENC) or from post-processing of cine images using feature tracking (FT) and tissue tracking (TT). However, it remains unclear whether strain values from different approaches are directly comparable, making the definition of universal reference values difficult. This study compared left ventricular (LV) strain assessed by FT, TT, and fSENC.

**Methods and results:**

We studied 240 individuals (183 patients recovered from coronavirus disease 2019 [COVID-19] and 57 age- and sex-matched healthy controls), who underwent standardized CMR including cine imaging and fSENC. LV global longitudinal (GLS), circumferential (GCS), and radial strain (GRS) were analysed using FT and TT; fSENC provided GLS and GCS. Global strain values differed significantly between all methods (*P* < 0.001). Agreement between FT and TT was high for GLS (bias −0.8%, *r* = 0.77) and moderate for GCS (bias −1.2%, *r* = 0.63), but poor for GRS (bias −6.0%, *r* = 0.37). Compared with fSENC, GLS showed moderate agreement for FT (bias 2.3%, *r* = 0.57) and TT (bias 3.0%, *r* = 0.59), while agreement for GCS was weaker. All approaches demonstrated excellent reproducibility. Post-COVID-19 patients showed a consistent but mild reduction in GLS compared with controls across all techniques (all *P* < 0.05).

**Conclusion:**

CMR strain imaging provides fast, reliable, and reproducible measurements. However, strain values are not directly interchangeable even between similar post-processing methods or when compared with dedicated sequences, highlighting the need for standardization and method-specific reference values.

## Graphical Abstract

**Graphical Abstract:**
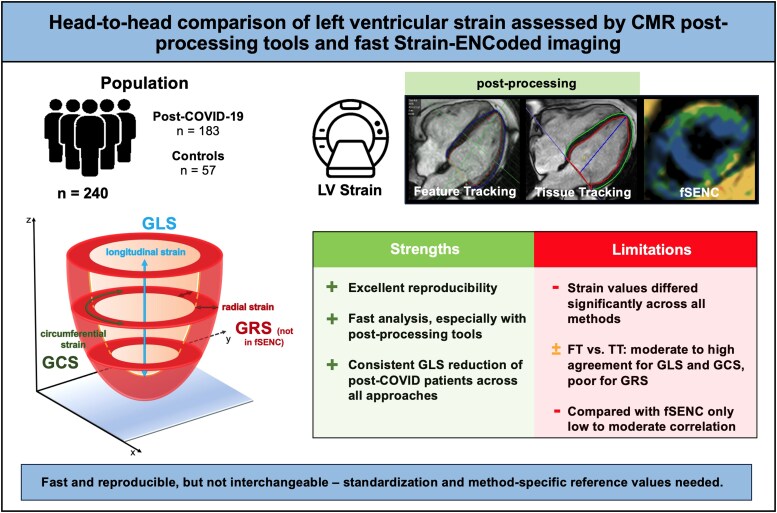

## Introduction

Cardiovascular magnetic resonance (CMR) imaging is a non-invasive, reliable, and reproducible modality for detecting subtle myocardial changes and is considered as the current gold standard for the assessment of cardiac morphology and function.^1,2^ CMR-based myocardial strain analysis has been shown to not only aid in diagnosis but also to provide more accurate risk prediction compared with conventional CMR parameters for various cardiac conditions, such as myocarditis or heart failure.^3,4^ It allows for the detection of early subclinical myocardial dysfunction, even without the use of a contrast agent.^5^ Despite its acknowledged benefits in various cardiac diseases, strain imaging is currently not supported for routine use in CMR or echocardiography according to clinical guidelines.^6,7^ The additional time required for strain analysis, as well as the need for specialized software solutions or dedicated CMR sequences, may contribute to its limited use.

Currently, many different imaging techniques and software tools are available for strain analysis in CMR. Along with methods such as tagging, SENC (Strain Encoded imaging) or DENSE (Displacement Encoding with Stimulated Echoes), which require dedicated MR sequences, post-processing feature tracking (FT) or tissue tracking (TT) have emerged as promising approaches. Both are applied to identical cine acquisitions and allow for retrospective strain measurements without additional CMR sequences. While strain measurements based on FT/TT demonstrate substantial agreement with echocardiography and the gold standard of MR tagging, especially for global strain values,^8–10^ they use different tracking algorithms, which may lead to systematic variations in strain results. Reference values have been published for adults and children for different strain approaches and tools.^11,12^ However, it remains unclear whether strain values provided by different approaches and vendors are directly comparable, with universal reference values still difficult to define.^13,14^ Furthermore, a reduction in strain analysis time using more automated software approaches integrated into commonly used workspaces may contribute to the implementation of strain analysis into clinical routine. In contrast to the post-processing approaches based on FT/TT, fast SENC (fSENC) is dependent on specific MR sequences but allows for reliable measurements of segmental strain as well.^15^ Recently, the fSENC technique has been shown to detect even small changes in myocardial strain for the early identification of patients with heart failure.^5^

This study aimed to compare different post-processing strain approaches and fSENC in a large, well-characterized cohort of individuals who all underwent a standardized CMR protocol. The study population consisted of individuals recovered from coronavirus disease 2019 (COVID-19) and healthy controls, in whom only minimal myocardial changes were previously observed.^16^ This setting allowed for a focused assessment of agreement and reproducibility, as well as an evaluation of method consistency in detecting subtle strain differences across techniques in a population without relevant pre-existing cardiac conditions.

## Methods

### Study subjects

This single-center, retrospective study consisted of 248 participants including 191 individuals recovered from COVID-19 and 57 age- and sex-matched healthy participants. Both cohorts had been part of earlier studies.^16,17^

Inclusion criteria of the post-COVID-19 subgroup required that participants had experienced severe acute respiratory syndrome coronavirus type 2 (SARS-CoV-2) infection at least four months prior to enrolment, confirmed by either a positive polymerase chain reaction for SARS-CoV-2 or serological detection of SARS-CoV-2 antibodies. Exclusion criteria were a known history of myocarditis before COVID-19 disease, cardiomyopathy, heart failure, obstructive coronary artery disease with prior coronary intervention or coronary artery bypass grafting, myocardial infarction or implantation of cardiac devices. Patients with non-obstructive coronary artery disease, as determined by coronary computed tomography angiography or invasive coronary angiography, were not excluded. All subjects underwent, prior to CMR, a comprehensive medical history, physical examination and 12-lead electrocardiogram (ECG). Detailed history of their COVID-19 illness, including concomitant symptoms and any hospitalizations, was collected using a questionnaire.

Additionally, a group of 57 age- and sex-matched, proven healthy volunteers of an established reference population was analysed retrospectively.^17^ They underwent a strict selection process including detailed medical history assessment, physical examination, 12-lead ECG, and comprehensive blood tests. Subjects with a history, signs, or symptoms of a cardiac disease were excluded. Single, well-controlled cardiac risk factors such as mild arterial hypertension were no exclusion criteria. Regular use of medications (except for vitamins, thyroid medication, or contraceptives) as well as any cerebrovascular or other relevant disease were exclusion criteria. Of note, CMR scans of the reference population were performed before the COVID-19 pandemic.

All participants gave written informed consent. The study was approved by the local ethics committee of the medical faculty of Heidelberg University (S-270/2021 and S-101/2019) and was conducted in accordance with the Declaration of Helsinki.

### Image acquisition and analysis

CMR examinations of post-COVID-19 subjects were performed using a mobile 1.5 Tesla clinical scanner (Ingenia, Philips Healthcare, Best, The Netherlands). The control group was scanned at a 1.5 Tesla or 3 Tesla scanner (Ingenia CX and Ingenia, Philips Healthcare, Best, The Netherlands).

The CMR protocol included balanced steady-state free precession (bSSFP) cine sequences (2-, 3-, and 4-chamber views, short-axis stack) with 8-mm slice thickness, 2-mm gap, and 35 phases per cardiac cycle. Typical acquisition parameters were: repetition time 2.8 ms and echo time 1.4 ms at 1.5 Tesla, 2.9 and 1.4 ms at 3 Tesla, with a flip angle of 60° and 45°, respectively. Prospective ECG gating was applied, and each image series was acquired during a 7–10 s breath-hold. Following cine acquisition, fSENC sequences were acquired in three short-axis (apical, mid-ventricular and basal) and three long-axis slices (2-, 3-, and 4-chamber views) using a single-heartbeat technique, as described previously.^15^ Since no contrast agent was administered, no late gadolinium enhancement was obtained.

All standard morphological and functional parameters were derived from short- and long-axis slices using commercially available workstations (IntelliSpace Portal V.12, Philips Healthcare, Best, The Netherlands) and dedicated CMR software (cvi42™, Circle Cardiovascular Imaging, Calgary, Alberta, Canada) in accordance with recent recommendations.^7^ Papillary muscles were included in the left ventricle (LV) blood volume.

### Strain analysis

**Figure 1:**
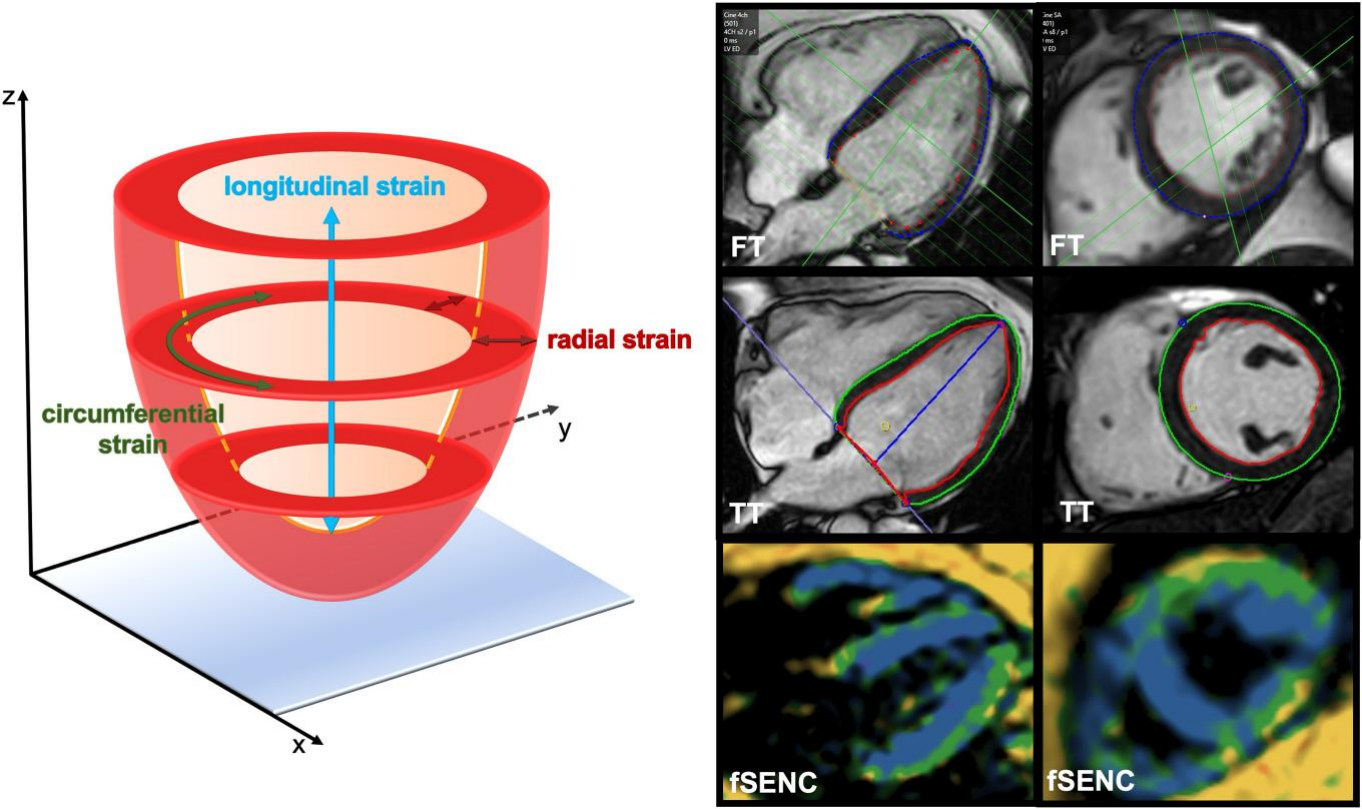
Schematic illustration of strain analysis for longitudinal, circumferential, and radial strain using different strain approaches. Left: The analysis of LV strain included longitudinal and circumferential as well as radial strain. Right: Examples of myocardial segmentation in the post-processing tools (FT, TT) and the colour-coded fSENC images of a four-chamber and a short-axis view. FT = feature tracking, fSENC = fast Strain-ENCoded imaging, LV = left ventricular, TT = tissue tracking.

FT/TT-based strain analysis was performed on bSSFP cine images. A fully automated FT-based post-processing software tool (Caas, Pie Medical Imaging BV, Maastricht, The Netherlands) integrated into the workstation (IntelliSpace Portal V.12) was used. This tool provided automated segmentation of the LV in end-diastole and tracking over the whole cardiac cycle. Only in case of insufficient segmentation/tracking, contours could have been corrected manually in end-diastole. The analysis allowed measurements of global and segmental LV longitudinal, circumferential, and radial strain (*Figure 1*). For the assessment of LV longitudinal strain, the FT tool only considered the 2- and 4-chamber view.

For comparison, a TT-based post-processing software tool (cvi42™) was used. This software allowed for both semi-automated as well as fully automated strain analysis. For a comprehensive comparison to the fully automated post-processing tool (Caas) regarding the time needed for strain analysis, we used the fully and semi-automated strain analysis for TT (cvi42™). For semi-automated strain analysis, endo- and epicardial borders were manually drawn in end-diastole, the tracking of the myocardium over the entire cardiac cycle was automatically conducted by the software and corrected manually if necessary.

For strain analysis using fSENC, dedicated software (MyoStrain 5.2.2, Myocardial Solutions, Inc., Morrisville, NC, USA) was used. As previously described, endo- and epicardial contours were drawn manually in three short axes for longitudinal strain and in three long axes for circumferential strain in end-systole, the radial strain cannot be assessed by fSENC.^17,18^

### Intra- and interobserver reproducibility

To assess intraobserver reproducibility, 20 randomly selected patients from the post-COVID-19 population were reanalysed by the same reader. For interobserver reproducibility, a second reader performed the analysis. To minimize a possible recall bias, the first and the second analyses were at least four weeks apart. All observers were blinded to prior analyses. The time for analysing each patient used for reproducibility measurements was documented to assess the average analysis time per patient for each strain approach.

### Statistical analysis

Data were tested for normal distribution using the Shapiro–Wilk test. For continuous parameters, mean ± standard deviation was used for parametric and median with interquartile range for non-parametric variables. For the comparison of continuous variables between two groups, (paired) Student’s *t*-test and Mann–Whitney *U* test were used as applicable.

For the comparison of more than two groups with normal distribution, repeated-measures ANOVA (analysis of variance) was used. Correlation between normally distributed variables was assessed using Pearson’s coefficient. Correlation coefficients were classified as reported before^19^: very high (*r* = 0.9–1.0), high (*r* = 0.7–0.9), moderate (*r* = 0.5–0.7), low (*r* = 0.3–0.5) and negligible (*r* = 0.0–0.3). Bland–Altman analysis was additionally performed to determine the mean bias and limits of agreement (LOA).^20^ Intra- and interobserver variability was calculated using the intra-class correlation coefficient (ICC with 95% CI) with a two-way random model with absolute agreement. Statistical analyses were performed using dedicated statistical software (MedCalc 23.0.9, Mariakerke, Belgium). A *P*-value < 0.05 was regarded as statistically significant.

## Results

The final study population consisted of 240 subjects (116 men, 124 women) with a mean age of 48.4 ± 14.3 years (*Table 1*). In total, eight subjects of the post-COVID-19 subgroup were excluded due to technical issues and poor image quality (*n* = 6), including cases in which sufficient strain analysis was impossible using FT (*n* = 2), fSENC (*n* = 1) or TT (*n* = 1), and due to aborted CMR scans (*n* = 2). In the subgroup of patients after COVID-19, CMR scans were performed a median of 395 (192–408) days after symptom onset. A detailed clinical characterization including symptoms during acute infection and persistent complaints, has been reported previously including hospitalization rates (*n* = 27, 14.8%) and persistent symptoms like general fatigue (42.9%), memory deficits (30.6%), and persistent exertional dyspnoea (13.7%) (see Supplementary data online, *Table S1*).^16^

**Table 1:** Demographics, CMR standard, and strain parameters assessed by different strain approaches of the total study population and the subgroups of post-COVID-19 patients and healthy individuals

	Total population (*n* = 240)	Post-COVID-19 cohort (*n* = 183)	Healthy individuals (*n* = 57)	*P*
**Demographics**				
Age, years	48.4 ± 14.3	48.4 ± 13.4	48.3 ± 17.1	0.962
Male gender, *n* (%)	116 (48.3%)	88 (48.1%)	28 (49.9%)	0.891
Weight, kg	76.8 ± 15.6	78.6 ± 15.8	71.0 ± 13.5	**0**.**001**
Height, cm	172.8 ± 9.4	173.0 ± 9.8	172.3 ± 7.7	0.568
BMI, kg/m²	25.6 ± 4.5	26.2 ± 4.7	23.8 ± 3.3	**<0**.**001**
BSA, m²	1.91 ± 0.22	1.94 ± 0.23	1.84 ± 0.20	**0**.**002**
**Cardiovascular risk factors or disease**				
Arterial hypertension, *n* (%)	49 (20.4%)	44 (24.0%)	5 (8.8%)	**0**.**002**
Dyslipidemia, *n* (%)	23 (9.6%)	18 (9.8%)	5 (8.8%)	0.813
Diabetes, *n* (%)	5 (2.1%)	5 (2.7%)	0	—
(History of) smoker, *n* (%)	58 (24.2%)	53 (29.0%)	5 (8.8%)	**<0**.**001**
Obesity, *n* (%)	32 (13.3%)	29 (15.8%)	3 (5.3%)	**<0**.**001**
CAD, *n* (%)	2 (0.8%)	2 (1.1%)	0	—
Heart rate, 1/min	66.2 ± 10.5	66.6 ± 11.1	64.9 ± 8.2	0.215
Atrial fibrillation, *n* (%)	0	0	0	—
**CMR standard parameters**				
LVEDV, mL	146.6 ± 33.7	148.7 ± 33.8	139.9 ± 32.8	0.086
LVESV, mL	57.4 ± 17.8	59.2 ± 18.0	51.6 ± 15.9	**0**.**004**
LVSV, mL	89.3 ± 19.1	89.5 ± 19.5	88.7 ± 18.0	0.781
LVEF, %	61.3 ± 5.5	60.6 ± 5.6	63.6 ± 4.7	**<0**.**001**
LV mass, g	90.7 ± 24.7	92.3 ± 24.3	86.3 ± 25.4	0.164
**CMR strain parameters**				
GLS_FT_, %	−17.3 ± 2.2	−17.1 ± 2.2	−17.9 ± 2.2	**0**.**023**
GLS_TT_, %	−16.5 ± 2.0	−16.2 ± 1.9	−17.7 ± 2.0	**<0**.**001**
GLS_fSENC_, %	−19.5 ± 1.8	−19.4 ± 1.8	−20.0 ± 1.6	**0**.**037**
GCS_FT_, %	−18.2 ± 2.8	−18.0 ± 2.9	−18.9 ± 2.7	**0**.**029**
GCS_TT_, %	−19.4 ± 2.8	−19.5 ± 2.9	−19.1 ± 2.0	0.218
GCS_fSENC_, %	−20.5 ± 1.8	−20.4 ± 1.5	−20.9 ± 1.4	**0**.**019**
GRS_FT_, %	28.6 ± 7.5	27.0 ± 5.7	33.9 ± 9.9	**<0**.**001**
GRS_TT_, %	34.0 ± 8.5	35.0 ± 9.1	33.6 ± 6.0	0.194

Values are mean ± SD or n (%). *P* values are for comparison of both subgroups. Significant *P* values (< 0.05) are in bold.

BMI = body mass index, BSA = body surface area, CAD = coronary artery disease, COVID-19 = coronavirus disease 2019, EDV = end-diastolic volume, EF = ejection fraction, ESV = end-systolic volume, fSENC = fast Strain-Encoded imaging, FT = feature tracking, GCS = global circumferential strain, GLS = global left ventricular strain, GRS = global radial strain, LV = left ventricular, SV = stroke volume, TT = tissue tracking.

No significant differences were observed between the post-COVID-19 group and healthy individuals with respect to age (48.4 ± 13.4 years vs. 48.3 ± 17.1 years, *P* = 0.962) and sex distribution (48.1% males vs. 49.9% males, *P* = 0.891) (*Table 1*). Arterial hypertension (24.0% vs. 8.8% in the control group, *P* = 0.002) and obesity (body mass index 26.2 ± 4.7 kg/m² vs. 23.8 ± 3.3 kg/m²; *P* < 0.001) were more prevalent in the post-COVID-19 group. LV ejection fraction (EF) was significantly higher in controls (63.6 ± 4.7% vs. 60.6 ± 5.6%, *P* < 0.001), with smaller LV end-systolic volumes (*Table 1*). Regarding LV strain, global longitudinal strain (GLS) was significantly less pronounced in the post-COVID-19 subgroup across all different strain approaches (post-COVID-19 cohort vs. healthy controls: GLS_FT_ = −17.1 ± 2.2% vs. −17.9 ± 2.2%, GLS_TT_ = −16.2 ± 1.9% vs. −17.7 ± 2.0%, GLS_fSENC_ = −19.4 ± 1.8% vs. −20.0 ± 1.6, all *P* < 0.05). Global circumferential strain (GCS) measured by FT and fSENC was less pronounced in the post-COVID-19 group, for GCS_TT_ no significant differences were observed. Less pronounced global radial strain (GRS) was observed in the post-COVID-19 group using FT, whereas differences in GRS_TT_ did not reach statistical significance. Compared with existing literature, GLS_FT_ was outside the reference range for normal strain values in only four post-COVID-19 patients with slightly impaired GLS_FT_ between −12.0% and −14.1%, using TT, nine patients were below the normal range (−11.9% to −14.0%).^21^ Of note, only two post-COVID-19 patients had reduced LVEF (<50%), both with normal strain (GLS −19.7%/−17.1%, GCS −19.4%/−21.5%). Conversely, impaired GLS was observed exclusively in patients with preserved LVEF (≥50%; range 50–66%).

Even in subgroup analysis, comparing post-COVID-19 patients and healthy individuals, both without arterial hypertension and obesity (body mass index < 30 kg/m^2^), significantly lower LV EF, less pronounced GLS, GCS, and GRS were found for most approaches in post-COVID-19 patients (see Supplementary data online, *Table S2*).

### Comparison of post-processing strain approaches

**Figure 2:**
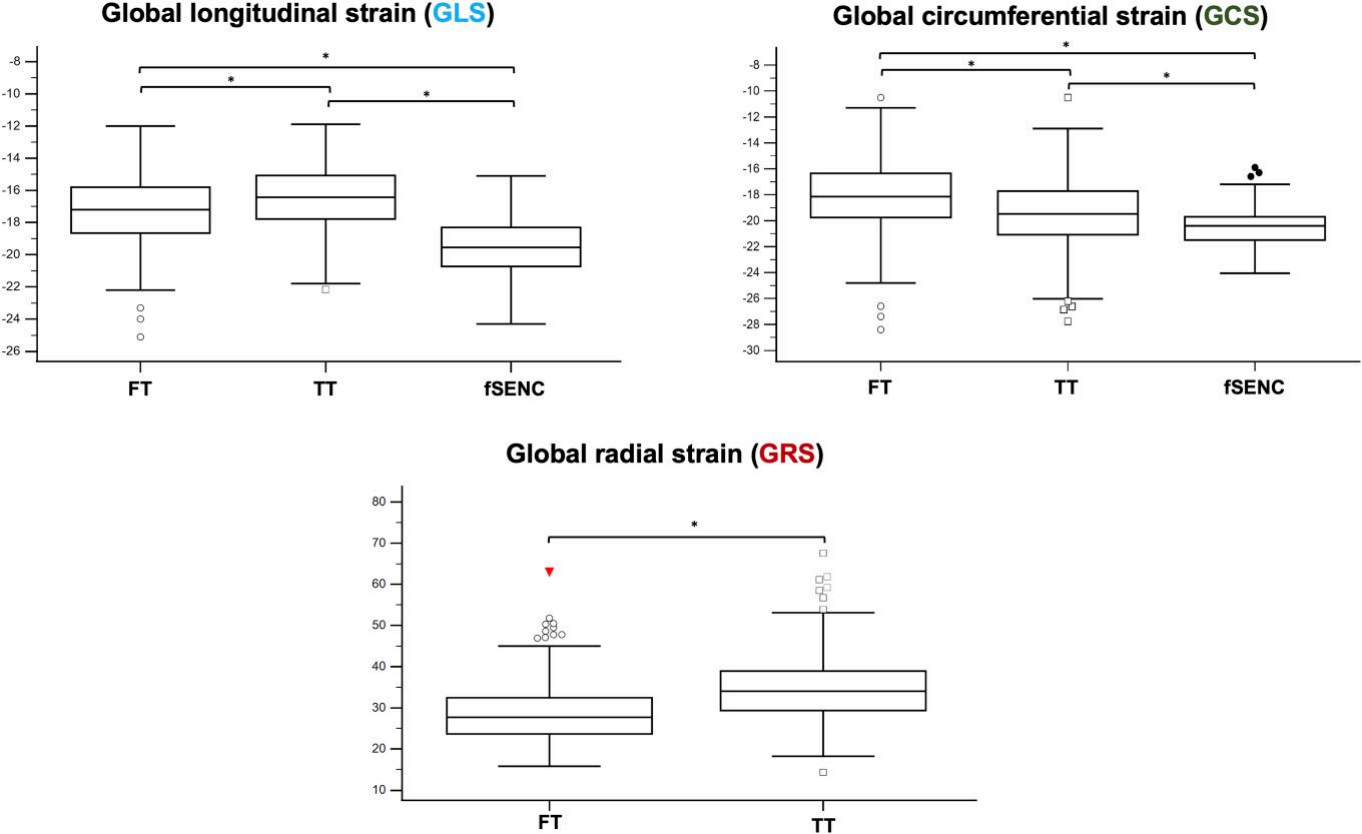
Comparison of GLS, GCS, and GRS between the different strain approaches. Box plots for the comparison of GLS, GCS, and GRS. Significant differences were observed for the strain parameters between all approaches. *P < 0.001. fSENC = fast Strain-ENCoded imaging, FT = feature tracking, GCS = global circumferential strain, GLS = global longitudinal strain, GRS = global radial strain, TT = tissue tracking.

**Figure 3:**
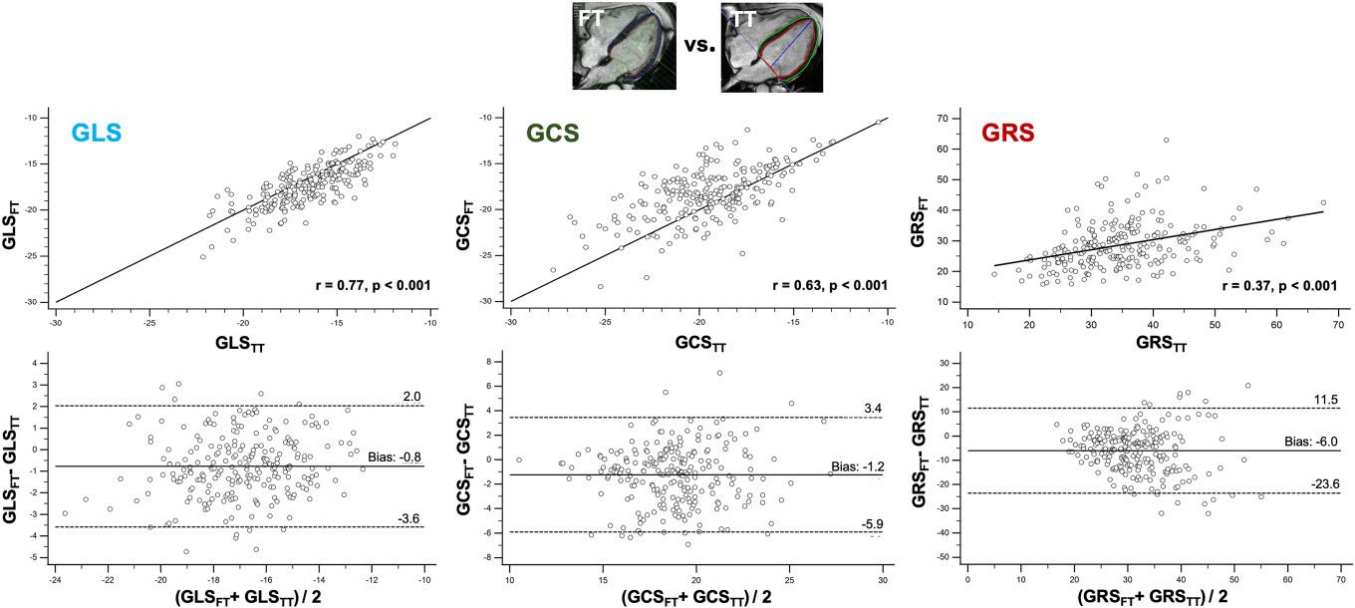
Comparison of global strain values derived by post-processing tools. Comparison (from left to right) of GLS, GCS, and GRS between both post-processing tools (FT and TT). Upper line: Linear regression analysis including Pearson correlation coefficient (*r*) and line of equality. Highest correlation was found for GLS (*r* = 0.77). Bottom line: Bland-Altman plot including bias (mean) and LOA (1.96 SD). Bias between both approaches was small for GLS and GCS, the bias for GRS was higher. FT = feature tracking, GCS = global circumferential strain, GLS = global longitudinal strain, GRS = global radial strain, SD = standard deviation, TT = tissue tracking.

**Figure 4:**
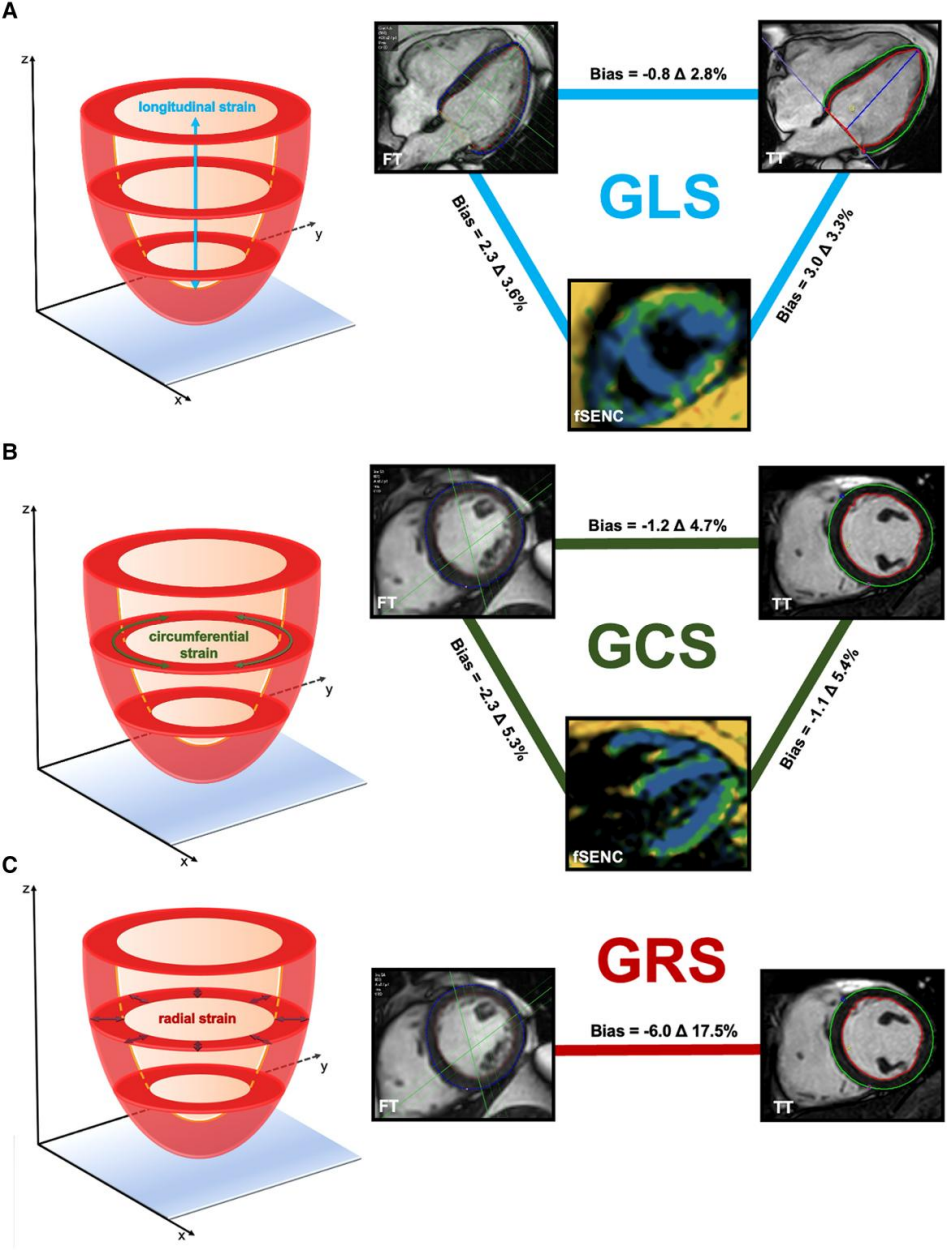
Illustration of bias and LOA for the assessment of GLS (*A*), GCS (*B*), and GRS (*C*) between the different approaches. Comparison of bias and LOA for GLS between different approaches. Bias and LOA were derived from Bland–Altman plot. LOA = 1.96 standard deviations. Δ = limit of agreement. fSENC = fast Strain-ENCoded Imaging, FT = feature tracking, GCS = global circumferential strain, GLS = global longitudinal strain, GRS = global radial strain, LOA = limits of agreement, SD = standard deviation, TT = tissue tracking.

Significant differences were observed in all global strain values when comparing the two post-processing strain approaches (*Table 1*, *Figure 2*). Specifically, GLS was more pronounced in FT (FT vs. TT: −17.3 ± 2.2% vs. −16.5 ± 2.0%, *P* < 0.001), GCS and GRS were less pronounced (FT vs. TT: GCS = −18.2 ± 2.8% vs. −19.4 ± 2.8%, GRS = 28.6 ± 7.5% vs. 34.0 ± 8.5%, both *P* < 0.001) compared with TT. The differences between the two post-processing strain algorithms for assessing global strain values are visualized in *Figures 3* and *4*. Bland–Altman analysis showed good agreement for GLS and GCS without systemic over- or underestimation. The bias was −0.8% for GLS, −1.2% for GCS, and −6.0% for GRS. The narrowest LOA was observed for GLS (Δ 2.8%); LOA for GCS (Δ 4.7%) and especially for GRS (Δ 17.5%) were significantly higher. The correlation between strain values assessed by FT and TT ranged from low for GRS (*r* = 0.37, *P* < 0.001) and moderate for GCS (*r* = 0.63, *P* < 0.001) to high for GLS (*r* = 0.77, *P* < 0.001).

### Comparison of post-processing strain approaches to fSENC

**Figure 5:**
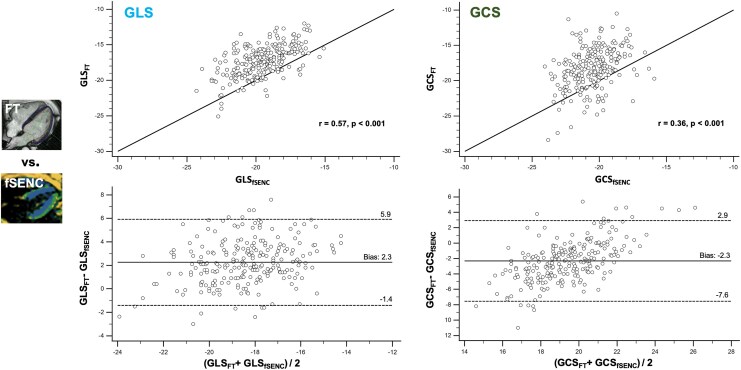
Comparison of global strain values derived by FT and fSENC. Comparison of GLS and GCS between the post-processing tool based on FT and fSENC. Upper line: Linear regression analysis including Pearson correlation coefficient (*r*) and line of equality. Highest correlation was found for GLS (*r* = 0.57). Bottom line: Bland-Altman plot including bias (mean) and LOA (1.96 SD). Bias between both vendors was small for both GLS and GCS. Using fSENC, GRS could not be assessed. fSENC = fast-Strain-ENCoded imaging, FT = feature tracking, GCS = global circumferential strain, GLS = global longitudinal strain, GRS = global radial strain, SD = standard deviation.

**Figure 6:**
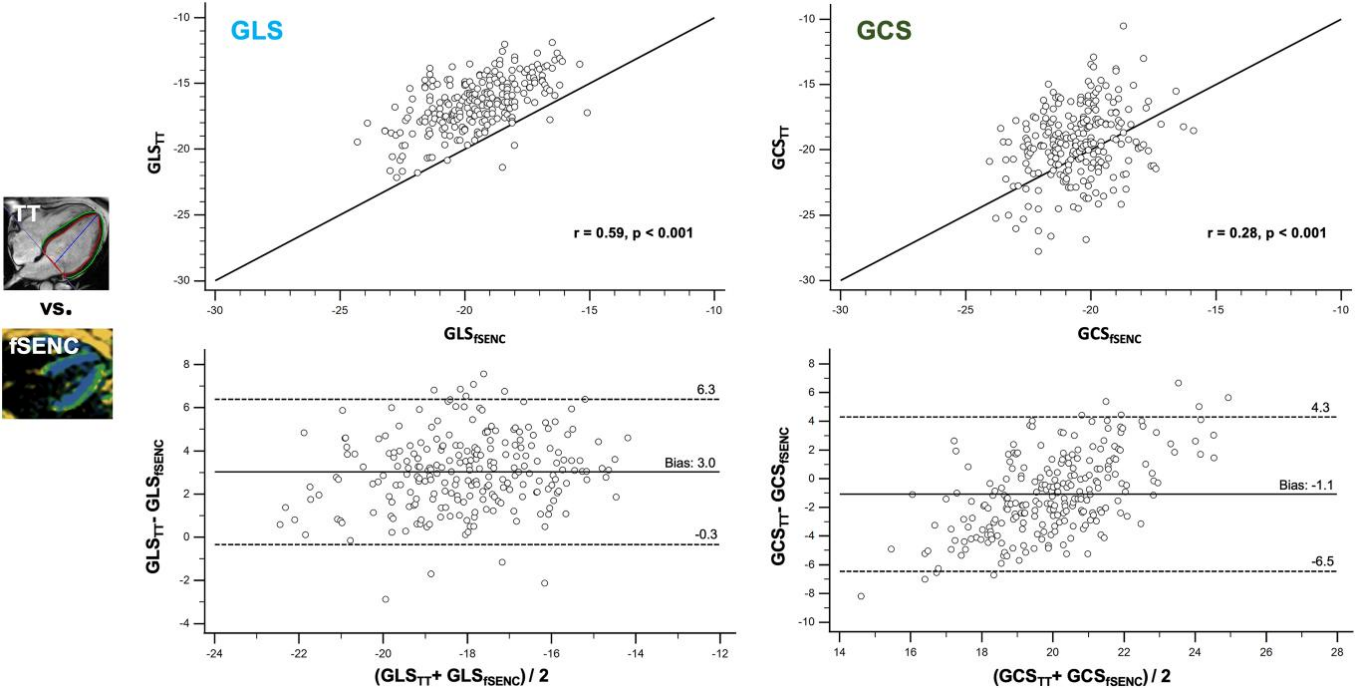
Comparison of global strain values derived by TT and fSENC. Comparison of GLS and GCS between the post-processing tool based on TT and fSENC. Upper line: Linear regression analysis including Pearson correlation coefficient (*r*) and line of equality. Highest correlation was found for GLS (*r* = 0.59). Bottom line: Bland–Altman plot including bias (mean) and LOA (1.96 SD). Bias between both vendors was small for GLS and GCS. GRS could not be assessed by fSENC. fSENC = fast-Strain-ENCoded imaging, GCS = global circumferential strain, GLS = global longitudinal strain, GRS = global radial strain, SD = standard deviation, TT = tissue tracking.

Compared with fSENC, global strain parameters derived from FT/TT were significantly lower (*Table 1*, *Figure 2*). The bias for GLS between FT and fSENC was 2.3% (Δ 3.6%), for GCS −2.3% (Δ 5.3%)—showing good agreement without systemic over- or underestimation (*Figures 4–6*). Between TT and fSENC, the bias was 3.0% (Δ 3.3%) for GLS and −1.1% (Δ 5.4%) for GCS. As shown in *Figures 5* and *6*, the correlation of global strain values between FT and fSENC was moderate for GLS (*r* = 0.57, *P* < 0.001) and low for GCS (*r* = 0.36, *P* < 0.001). The correlation between TT and fSENC was almost similar [GLS (*r* = 0.59, *P* < 0.001), GCS (*r* = 0.28, *P* < 0.001)].

### Feasibility

Due to insufficient tracking quality, strain analysis was not possible in two patients using FT (2/248 = 0.8%), in one patient using fSENC and one using TT (each 1/248 = 0.4%). The fully automated strain approach based on FT ensured a significant time saving compared with a semi-automated approach (fully automated FT vs. semi-automated TT: 343 ± 96 s vs. 434 ± 124 s, *P* = 0.013), despite multiple corrections when the tracking was insufficient in the approaches. The fully automated TT was even faster compared with fully automated FT or semi-automated TT (fully automated TT: 249 ± 83 s, all *P* < 0.001 for comparison to the other approaches). Compared with the analysis of fSENC, there was no significant time saving of fully automated FT (fSENC vs. FT: 368 ± 43 s vs. 343 ± 96 s, *P* = 0.191), the difference to fully automated TT was significant (fSENC vs. fully automated TT: 368 ± 43 s vs. 249 ± 83 s, *P* < 0.001). However, additional time for the planning and the acquisition of fSENC sequences must be considered, leading to a prolongation of CMR scanning time for fSENC.

### Intra- and interobserver reproducibility of global strain values

Both post-processing approaches and fSENC featured good intra- and interobserver reproducibility for all global strain parameters (*Table 2*): the ICC for intraobserver variability for FT ranged between 0.95 (95% CI: 0.88–0.98) for GRS and 0.99 (95% CI: 0.96–0.99) for GLS, and the ICC for interobserver variability between 0.88 (95% CI: 0.01–0.97) for GCS and 0.95 (95% CI: 0.68–0.98) for GLS. For TT, intraobserver ICC ranged from 0.96 (95% CI: 0.90–0.98) for GLS to 0.98 (95% CI: 0.95–0.99) for GRS, and interobserver reproducibility ranged from 0.97 (95% CI: 0.90–0.99) for GCS to 0.97 (95% CI: 0.90–0.99) for GRS. Regarding fSENC, intraobserver ICC was 0.97 (95% CI: 0.89–0.99) for GCS and 0.98 (95% CI: 0.88–1.00) for GLS, at interobserver level it was 0.84 (95% CI: 0.27–0.97) for GCS and 0.98 (95% CI: 0.94–0.99) for GLS.

**Table 2:** Reproducibility of GLS, GCS and GRS using different strain approaches

	Intraobserver reproducibility ICC (95% CI)	Interobserver reproducibility ICC (95% CI)
**FT**		
GLS	0.99 (0.96–0.99)	0.95 (0.68–0.98)
GCS	0.98 (0.94–0.99)	0.88 (0.01–0.97)
GRS	0.95 (0.88–0.98)	0.91 (0.38–0.98)
**TT**		
GLS	0.96 (0.90–0.98)	0.99 (0.99–1.00)
GCS	0.98 (0.94–0.99)	0.97 (0.90–0.99)
GRS	0.98 (0.95–0.99)	0.97 (0.88–0.99)
**fSENC**		
GLS	0.98 (0.88–1.00)	0.98 (0.94–0.99)
GCS	0.97 (0.89–0.99)	0.84 (0.27–0.97)

CI = confidence interval, fSENC = fast Strain-Encoded imaging, FT = feature tracking, GCS = global circumferential strain, GLS = global left ventricular strain, GRS = global radial strain, ICC = intra-class-correlation coefficient, TT = tissue tracking.

## Discussion

This study systematically compared LV strain assessed by three different CMR-based approaches in a well-characterized cohort of 240 individuals, including 183 patients recovered from COVID-19 and 57 healthy controls, all examined using a standardized imaging protocol. The main findings were: (1) Strain values were not interchangeable between different techniques, even when comparing post-processing tools. (2) Fully automated post-processing strain tools based on FT/TT provided fast and reproducible global strain measurements. (3) GLS was slightly reduced in post-COVID-19 patients across all methods compared with healthy controls.

### Comparison and reproducibility of different strain approaches

Over the past decade, numerous post-processing tools have been developed to assess myocardial strain. Many studies have evaluated the different strain approaches, with FT/TT techniques showing good reproducibility, especially at the global level.^9,22^

Global strain values obtained with fSENC (GLS, GCS) were significantly more pronounced than those from post-processing approaches (FT/TT). Comparing FT to TT, we observed a good correlation with low bias for GLS and GCS. GRS showed an overall lower correlation and greater bias between both post-processing tools. A similar pattern was seen when comparing GLS and GCS to fSENC. The results were similar to those by Siry *et al.* and Valdés *et al.*, both using TT and fSENC for strain analysis,^13,14^ while agreement was lower than in the studies by Bucius *et al.* or El-Saadi *et al.*^23,24^ likely reflecting software and sample size differences. Radial strain showed greater variations between FT and TT, likely due to differences in myocardial contouring and the axes used for measurement, as radial deformation can be derived from both long- and short-axis views depending on the software.^25^ In terms of reproducibility, correlation and systematic bias, GLS consistently demonstrates the highest reliability, irrespective of the strain approach applied.

Since the exact algorithms of FT/TT-based software are proprietary and not published, the reasons for differences cannot always be determined. According to the recommendations for standardization of deformation imaging, none of the post-processing tools used the average of peaks of the segmental curves.^26^ In both FT (Caas) and TT (cvi42), global strain is calculated as the peak value of the averaged strain curves from the entire myocardial contour. This approach, previously described for TT,^27^ was also confirmed by the developer of our FT tool (Caas), indicating comparable algorithmic principles between the tools. However, despite these similarities, systematic deviations persisted, suggesting that even small variations in contour definition, tracking methodology, or post-processing steps can lead to clinically relevant discrepancies.

Reference values from Kawel-Böhm *et al.*^21^ were used for FT and TT, both post-processing techniques. Nevertheless, although applied to the same cine data, FT and TT yielded systematically different strain values, highlighting the need for method-specific reference ranges. For fSENC, normal data remain scarce and were adopted from Weise Valdés *et al.*^14^

Routine clinical implementation of strain imaging requires further standardization and evaluation of reference values across different approaches and vendors.^28,29^

### Perspectives of CMR strain imaging

Post-processing strain software that is integrated into routine clinical workflows allows for fast and reproducible assessment of LV strains. These integrated tools, along with automated myocardial segmentation, significantly reduce strain analysis time compared with semi-automated approaches. In the past years, strain analysis software has significantly improved, particularly regarding the time reduction of a comprehensive LV strain analysis from over 30 min,^30^ to about 10–20 min in the past years,^28^ to less than 5 min, including quality checks and corrections.

At the same time, recent strain software quantifies myocardial strain with excellent intra- and interobserver reproducibility for global strain values. While reliable echocardiographic strain measurements depend on several factors such as good image quality, CMR-assessed LV strain is not limited by the patient’s acoustic window and benefits from excellent intrinsic blood-tissue contrasts.^31^ In addition, sequence-dependent techniques such as tagging or fSENC allow reproducible analysis of segmental strains.

### LV strain in the post-COVID-19 cohort

The post-COVID-19 cohort analysed in this study was previously characterized in a separate publication focusing exclusively on fSENC strain analysis.^18^ In contrast, the present work systematically compares multiple CMR-based strain techniques, including FT and TT, in a well-defined cohort of recovered COVID-19 patients and matched controls, thereby providing additional insights into consistency and reproducibility of different strain approaches.

In line with previous CMR studies, we observed slightly reduced global strain parameters in post-COVID-19 patients compared with controls.^32,33^ While statistically significant, the clinical relevance is uncertain. The post-COVID-19 group included a more heterogeneous, real-world population with a higher prevalence of arterial hypertension and obesity—both known to affect strain values.^34,35^ However, it’s noteworthy that even in subgroups without these risk factors, significant differences in LV strain persisted for all different strain approaches compared with controls. Importantly, most values remained within published normal ranges,^21^ in line with earlier CMR studies reporting only subtle differences between patients and controls after COVID-19.^32,33^

Although differences in LV strain between the post-COVID-19 cohort and healthy controls were small, GLS was consistently lower across all methods, highlighting the robustness of CMR-based strain analysis in detecting even subtle myocardial alterations.

### Limitations

This study has several limitations. First, the post-COVID-19 population was heterogeneous, and disease information was obtained retrospectively through patient history, partly via self-reported questionnaires. Pre-existing cardiac conditions or comorbidities were not systematically excluded, so undiagnosed findings may have been present in asymptomatic individuals. In contrast, the control group consisted of thoroughly screened healthy volunteers.

Secondly, the 3-chamber view was not part of the longitudinal strain analysis in the FT-based Caas tool, which may have contributed to minor differences of GLS between FT and TT, even in healthy subjects. Consequently, the anteroseptal and inferolateral segments including the LV outflow tract were excluded from GLS calculation, which may lead to overestimation in patients with regional wall motion abnormalities (e.g. post-myocardial infarction).

Thirdly, manual correction of strain contours in Caas was limited to end-diastole. As a result, two patients had to be excluded due to poor tracking quality that could not be corrected adequately.

Fourthly, a subset of healthy controls (*n* = 11) underwent CMR at 3 Tesla, whereas the majority was scanned at 1.5 Tesla. The comparability of LV strain—especially with fSENC—between different field strengths remains unclear.

Fifth, MR tagging, the reference standard for strain quantification, was not included due to the time-optimized CMR protocol.

The additional time for fSENC planning and acquisition was not measured in this study.

## Conclusions

CMR post-processing tools provide fast, reliable and reproducible strain measurements. Subtle global strain changes can be accurately detected across different techniques. More automated post-processing tools may further facilitate the integration of myocardial strain analysis into clinical practice. However, strain values derived from different approaches were not interchangeable, even between similar post-processing tools, highlighting the need for standardization and method-specific reference values. Across all techniques, GLS showed a consistent mild impairment in the post-COVID-19 subgroup, while most values remained within the normal range.

## Supplementary Material

qyag014_Supplementary_Data

## Data Availability

The datasets used and/or analysed during the current study are available from the corresponding author on reasonable request.

## Lead author biography

Andreas Ochs is a cardiologist and researcher at the University Hospital Heidelberg, Germany, with long-standing expertise in cardiovascular magnetic resonance (CMR). He has been involved in CMR research since 2014, primarily focusing on strain imaging techniques. His scientific work explores the prognostic implications of myocardial strain in cardiomyopathies and other cardiac conditions, and the clinical utility of fast strain-encoded imaging (fSENC) for non-invasive ischaemia detection without pharmacological stress. Dr. Ochs became a board-certified cardiologist in 2024 and holds the Level III certification in CMR from the German Society of Cardiology (DGK).
