# Supplementary material for: Head-to-head comparison of left ventricular strain assessed by CMR post-processing tools and fast strain-ENCoded imaging
Source: Eur Heart J Imaging Methods Pract. 2026 Jan 23;4(1):qyag014. doi: 10.1093/ehjimp/qyag014 (PMC12877874; doi:10.1093/ehjimp/qyag014)
Supplement: qyag014_Supplementary_Data [file qyag014_supplementary_data.docx]

**Supplemental Data**

**Table 1.** **Clinical Characteristics of the post-COVID-19 cohort (n=183).**

Values are median (IQR) or n (%).

|  |  |  |  | |  |
| --- | --- | --- | --- | --- | --- |
| **Time since COVID-19, d** | 395 (192-408) | | |  | |
| **Acute COVID-19 symptoms** |  | | | **Persistent Symptoms** | |
| Fever, n (%) | 106 (57.9%) | | | 2 (1.1) | |
| Cough, n (%) | 114 (62.3%) | | | 15 (8.2) | |
| Headache, n (%) | 121 (66.1%) | | | 2 (1.1) | |
| Melalgia, n (%) | 120 (65.6%) | | | 20 (10.9) | |
| Loss of smell/taste, n (%) | 109 (59.6%) | | | 24 (13.1) | |
| Chest pain, n (%) | 53 (29.0%) | | | 18 (9.8) | |
| Dyspnea, n (%) | 67 (36.6%) | | | 25 (13.7) | |
| Edema, n (%) | 9 (4.9%) | | | 5 (2.7) | |
| Impaired performance, n (%) | 141 (78.3%) | | | 63 (34.4) | |
| Syncope, n (%) | 4 (2.2%) | | | 0 (0) | |
| Palpitations, n (%) | 36 (19.7%) | | | 25 (13.7) | |
| Memory disturbance, n (%) | 75 (41.2%) | | | 56 (30.6) | |
| Other symptoms, n (%) | 63 (34.8%) | | | 19 (10.3) | |
| **COVID-19 severity** |  | | |  | |
| Hospitalization, n (%) | 27 (14.8%) | | |  | |
| Days of hospitalization, d | 8 (5-13) | | |  | |
| IMC/ICU, n (%) | 13 (7.1%) | | |  | |
| Days of IMC/ICU stay, d | 4 (1-7) | | |  | |
| Oxygen needed, n (%) | 14 (7.7%) | | |  | |
| Duration of oxygen, d | 6 (4-9) | | |  | |
| Invasive ventilation needed, n (%) | 3 (1.6%) | | |  | |
| Duration of ventilation, d | 14 (12-25) | | |  | |

COVID-19 = coronavirus disease 2019, ICU = intensive care unit, IMC = intermediate care unit.

**Table 2. Comparison of subgroups without arterial hypertension and obesity.**

Values are mean ± SD. P values are for comparison of both subgroups. Significant p values (< 0.05) are in bold.

|  | **Post-COVID-19 cohort (n=126)** | **healthy individuals**  **(n=49)** | **p** |
| --- | --- | --- | --- |
| **LVEF (%)** | 59.9 ± 5.4 | 63.8 ± 4.3 | **<0.001** |
| **GLS_FT_ (%)** | -17.1 ± 2.1 | -18.1 ± 2.2 | **0.008** |
| **GLS_TT_ (%)** | -16.2 ± 1.8 | -17.8 ± 1.9 | **<0.001** |
| **GLS_fSENC_ (%)** | -19.5 ± 1.8 | -20.0 ± 1.5 | 0.057 |
| **GCS_FT_ (%)** | -18.0 ± 2.8 | -19.0 ± 2.5 | **0.026** |
| **GCS_TT_ (%)** | -19.4 ± 2.9 | -19.3 ± 2.0 | 0.767 |
| **GCS_fSENC_ (%)** | -20.6 ± 1.4 | -21.1 ± 1.3 | **0.026** |
| **GRS_FT_ (%)** | 26.9 ± 5.5 | 32.9 ± 12.0 | **<0.001** |
| **GRS_TT_(%)** | 34.6 ± 8.9 | 34.1 ± 5.9 | 0.747 |

COVID-19 = coronavirus disease 2019, fSENC = fast Strain-ENCoded Imaging, FT = feature tracking, GCS = global circumferential strain, GLS = global left ventricular strain, GRS = global radial strain, LVEF = left ventricular ejection fraction, TT = tissue tracking.
